# Medical Expert Testimony1Read by invitation at the 82d annual meeting of the Medical Society of the County of Erie, January 13, 1903

**Published:** 1903-03

**Authors:** Carey D. Davie

**Affiliations:** Salamanca, N.Y., Surrogate of Cattaraugus County


### Medical Expert Testimony.[1]

By Hon. CAREY D. DAVIE, Salamanca, N.Y.,
Surrogate of Cattaraugus County.

MEDICAL expert testimony has become so important a factor in our civil and criminal jurisprudence, that the principles governing its production and application are subjects of interest to every reputable physician and surgeon. The ordinary witness speaks of tangible facts coming under his observation; his province is simply that of narration; the value of his evidence depends upon his integrity and the accuracy of his memory; whereas the expert speaks of conclusions, the result of careful study and investigation, beyond the comprehension of the ordinary observer. The value of such evidence depends upon the honor, experience and sound judgment of the expert.

We are often confronted with the spectacle of prominent experts expressing and vehemently defending opinions diametrically opposed—a fact that has contributed much in discrediting this species of evidence. I was recently engaged in hearing a case as referee, where the action was brought by a woman to recover damages for serious injuries claimed to have been sustained in consequence of a fall upon a defective walk. The plaintiff was concededly suffering from chronic spinal irritation with its usual attendant serious complications. Three medical experts, gentlemen of pronounced ability and undoubted integrity, unhesitatingly attributed her condition to the injury from the fall; three other experts of equal professional attainment asserted with the same degree of certainty, that such condition was the result of extreme illness on the part of the plaintiff through a complicated case of obstetrics two years before her fall. The measure of damages necessarily depended upon a correct determination of this question and the trial court, unskilled in these particular lines of investigation, was compelled to decide between such conflicting and irreconcilable expert testimony.

It has often come under my observation in connection with proceedings for the probate of wills, that an array of distinguished specialists unhesitatingly pronounce the testator of sound and disposing mind at the time of the execution of the will; while an equal number of experts, having had the same opportunity for forming a correct conclusion, unqualifiedly

1. Read by invitation at the 82d annual meeting of the Medical Society of the County of Erie, January 13, 1903.

testify to total lack of testamentary capacity. The same situation is observed in the records of almost every important trial where it became necessary to inquire into the cause of death, cases of homicide, suicide or accidental death; in cases of birth of infants where it was important to determine whether such infant had been born alive; in criminal prosecutions for rape, abortion and bastardy; in an innumerable variety of actions to recover damages for personal injuries where the nature, extent and permanency of such injury were involved; and in actions against members of the medical profession for malpractice involving the degree of skill usual and that actually used in the case under investigation. This state of affairs has led to many stinging criticisms upon medical expert testimony, not only from ordinary observers, but through the public press and often from high and authoritative sources. As great a jurist as Lord Kenyon, has said that skilled witnesses come with such a bias on their minds to support the cause in which they are embarked, that hardly any weight should be given to their evidence.

Judge Davis, of the Supreme Court of Maine, in his opinion in the famous Neil case says:

If there is any kind of testimony that is not only of no value, but even worse than that, it is, in my opinion, that of medical experts; they may be able to state the diagnosis of a disease more learnedly, but upon the question whether it had at a given time, reached such a stage that the subject of it was incapable of making a contract or irresponsible for his acts, the opinions of his neighbors, of men of good common sense, would be worth more than all the experts in the country.

Judge Finch of the Court of Appeals, in the case of Griswold vs. N. Y. C. & H. R. R. Co., says: "Medicine is very far from being an exact science; at the best its diagnosis is little more than a guess enlightened by experience."

Justice Rumsey, of the Appellate Division, says: "The sole difference between him (the expert witness) and an advocate being that he makes his argument under oath, and that he endeavors to add the weight of the oath to the opinion of the advocate."

Justice Adams, of Canandaigua, says: "Expert witnesses are far more anxious to destroy each other than to elucidate the particular question in issue."

Wharton, in his noted work on Evidence, after observing that expert testimony when first introduced was regarded with great respect, because an expert was then viewed as the representative of a science of which he was a professor, giving the impartial conclusions of such science, continues:

> Two conditions have combined to produce a material change in this relation. In the first place it has been discovered that no expert, no matter how learned and incorrupt, speaks for his science as a whole; few specialties are so small as not to be torn by factions; and often the smaller the specialty the more bitter, inflaming, and distorting are the animosities by which these factions are possessed; particularly is this the case in matters psychological, in which there is no hypothesis so monstrous that an expert can not be found to swear to it and defend it with vehemence. *Nihil tam absurdo*, which being literally translated means that there is nothing so absurd that the philosophers wont say it.

Now, I do not quote these expressions with any degree of personal approval, but simply to show that criticism does prevail, in order that we may, if possible, ascertain the cause and determine the best method of refuting such criticism if unjust, or eradicating the occasion for it if well founded. In attempting this task I shall make certain general observations upon our existing system of expert testimony, then suggest certain changes which I believe would be beneficial.

The practical value of the testimony of an expert depends largely upon his personal appearance and conduct as a witness. No one can successfully meet these requirements without possessing a comprehensive understanding of what expert testimony really is, his duties and obligations, rights and privileges in regard to the same. If a physician is called to testify as an expert in some particular case, it does not imply that he is retained as special counsel in such litigation; it affords no reason why he should become unduly solicitous for the success of the party by whom he is called; it does not mean that the physician has become substituted as a party of record in the case; the success of the party for whom he appears does not necessarily establish a vindication of his views and theories, nor the defeat of such party condemnation of his professional judgment. All the expert is called upon to do is to testify to what he may actually know regarding the matter in controversy, and conscientiously express his opinion based upon such knowledge. Having done this his functions in the case are terminated; the lawyers will look after the conduct of the litigation, and the defeated party and not the expert will be called upon to pay the costs. Jurors are usually acute in discerning the motives and interest of a witness, and the moment it is discovered that the expert is strongly biased in favor of either party his evidence loses much of its force and weight.

Again, testifying as an expert is in no sense a species of dress parade for the doctor; it is not designed as a public exhibition of the expert's attainments and qualifications; he is not produced for the purpose of edifying his hearers with professional gymnastics, nor is his cross-examination intended as a wrestling match between the witness and counsel. A judicial investigation is not a Vanity Fair; there are no settings of red fire or pyrotechnics in connection with the testimony of the conscientious expert, whose only desire is to aid the machinery of justice in determining where the truth of the controversy actually abides. Many an expert has met his Waterloo because of his attempts at wit and his efforts to irritate the counsel; the lawyer always has the advantage; if you wish to "get even" with him wait until you have him upon the operating table for an amputation or to remove the appendix.

No physician should permit himself to be used as an expert unless he is thoroughly conversant with the subject matter under investigation; the inefficient witness either produces incalculable harm, or makes himself ridiculous by an exposure of his charlatanry through the instrumentality of cross-examination. It is injudicious for the doctor to deal in fallacies because he speaks of subjects beyond the comprehension of the ordinary observer; his bauble of error may be punctured, showering him with confusion. A physician of a rural county was recently called to testify to the insanity of the defendant in a criminal prosecution; in point of experience he was no better qualified as an alienist than a village blacksmith; having testified that the defendant was a "monomaniac" he was requested to explain upon cross-examination the technical meaning of the term "mono" as used by him; he said that "mono" meant a part; that insanity was an affection of the mind, but whether a monomaniac was a man whose mind was partly affected or a part of whose mind was affected, he could not tell; but he finally succeeded in defining a monosyllable as a word of two and a dissyllable as a word of three syllables.

The expert should avoid the use of technical terms; he should give his testimony in the English language; nothing is more absurd than for the expert who is called for the purpose of elucidating some proposition pertaining to his profession, attempting to do so in phrases absolutely meaningless to the jury and often to his honor upon the bench. There are but few medical terms or expressions not susceptible of translation into language within the comprehension of the man of ordinary experience. The functions of an expert are those of a demonstrator and not a mystifier; it is clear, practical elucidation and not legerdemain that the occasion requires. The expert's statement that a certain person whom he was describing had a small excrescence or tumor of the skin, consisting of hypertrophied cutaneous papillæ, bound together with hard scaly epithelium, might be technically correct, and yet it would hardly convince the ordinary juror that such person had simply a wart upon his nose. It is as easy to speak of water as aqua pura; of whiskey as spirits frumenti; of salt as chloride of sodium.

The task of the expert upon his direct examination will usually be free from embarrassment; he will ordinarily have an opportunity of conferring with the counsel calling him, before his examination, regarding the general features of his evidence. This is not only a proper but a necessary preliminary; no careful lawyer attempts to examine an expert without previous consultation; and the doctor should not consent to testify as an expert, without being fully appraised in advance of the general trend of his examination. The examination in chief is often unproductive, and sometimes farcical, because the counsel and the expert are not "in tune"; they misunderstand each other; meaningless questions elicit confusing and unfortunate answers; but to the lawyer who possesses a clear conception of what he desires to prove, and to the expert who has had an opportunity of logically arranging his thoughts and concisely formulating his manner of expression, the pathway of examination in chief will be beset with but few perils. It is the cross-examination which tests the mettle of the expert; in order to successfully pass this ordeal, the expert should thoroughly understand the methods or characteristics of his cross-examiner; he will belong to one of three classes; one can easily diagnosticate his case and determine to which class he belongs. He may be the professional "bulldozer" who glares at you, talks in a loud tone, treats you as if you had insulted him, frequently admonishing you to "remember, sir, you are under oath," bristling with pointed and irritating interrogatories, like the quills of the proverbial fretful porcupine. Have no fear of him; it is the lightning and not the thunder that kills; self-possession, unruffled good nature, and short and decisive answers on your part will soon place your would-be tormentor entirely *hors de combat.*

Again, you will be confronted with counsel who has evidently primed himself for your cross-examination by a somewhat careful study of the medical authorities bearing upon the subject under investigation; under the pretext of testing your competency as an expert, he will subject you to an examination reminding you of your experiences while being examined for your degree. For example, if the case under investigation be one of arsenical poisoning, he will insist on your describing arsenic, its chemical formula, its color, specific gravity, atomic weight, vapor density and the process of its preparation by sublimation; he will call for a detailed explanation of Marsh's test; he will inquire as to the most effective chemical antidote for arsenic, and if you answer that it is the hydrated sesquioxide of iron, he will ask you to explain the reason why! his object being all the time, not to develop any information germane to the issue, but to discredit you; to find some opening in your armor; to compel you to confess your ignorance and, incidentally, to show the jury how much more efficient he is, who is simply a lawyer, than you are, though posing as an expert.

This line of cross-examination may be somewhat troublesome; the court can hardly interfere, even upon the objection of the opposing counsel; the only satisfactory method of fortifying yourself against such an attack is by absolute familiarity with all the details of the subject under investigation. If you are unable to answer any particular interrogatory frankly acknowledge your inability; never hazard a guess; an erroneous answer however unimportant or foreign to the issue, will be detected and used to discredit you; you have an absolute right to say you do not know; the court and jury will respect your candor and you will thereby wrest from your examiner the weapon he is seeking to use against you.

Again, you will meet the cool, deliberate and thoroughly well-informed cross-examiner. Counsel appearing for corporations and wealthy litigants are usually of this class; they have had extended experience in this line of cross-examination; they are competent to grasp the full import of your testimony; they will treat you with the utmost respect and gain your confidence if possible; they will carefully and craftily lead you along from one proposition to another, and before you realise your peril will elicit a line of answers absolutely irreconcilable with your previous testimony. They will dismiss you with all due deference, but you may depend upon it, they will skilfully remove your professional cuticle in the final argument before the jury. Dealing with this class of cross-examiners calls for the utmost tact, coolness, deliberation and absolute frankness on the part of the expert; a thorough familiarity with the subject and an unwavering fidelity to the absolute truth will ordinarily afford you a sufficient shield and protection.

The attendance of an expert upon the trial of an action is accomplished through the instrumentality of a subpena and the payment of the statutory witness fees. The impression sometimes prevails that an expert is justified in refusing to obey the requirements of the subpena until he is paid the fees of an expert; this impression is erroneous and if persisted in might land its advocate in jail for contempt of court. The physician must obey the mandate of the court and his rights as an expert will be guarded in the progress of the proceedings.

It has been a subject of interesting discussion whether experts should receive any other compensation than that prescribed for ordinary witnesses. The decisions upon this subject are not uniform, the courts of many states holding, that it is so far a necessary part of the judicial system that the expert may be called upon to express the results of his knowledge, skill and experience without extra compensation. The courts of other states have recognised the claim and supported it by decisions, that the knowledge and skill acquired by study and experience along special lines become a species of property, and the enjoyment and possession of the same fully protected by the constitutional guarantees. In this state the proposition is well settled, that one can not be compelled to testify as an expert until he has been compensated as such; then the proper course to be pursued by the physician who has been subpened is to obey the mandate and arrange, if possible, with the counsel calling him as to his fees; or, when asked to give expert evidence, decline to do so until proper compensation has been provided for. It is the duty of the court to protect the expert in the exercise of this right.

It is a proper subject of cross-examination as to what compensation has been paid or promised the expert as a witness. In one case it was held that where the expert admitted that his compensation was contingent upon the result of the litigation, he was disqualified from testifying at all. The subject of compensation being a proper matter of cross-examination for the purpose of developing the interest of the witness, he will avoid embarrassment by frankly answering every proper question put to him upon that subject.

It is the province of the court to determine the question of the competency of a witness presented as an expert; such inquiry, however, is governed by well-defined rules and principles. If it appear upon such examination upon the question of competency that the witness is duly licensed to practise medicine, that fact alone entitles him to be sworn as an expert and the court can not exclude his evidence, though he may never have had a case similar to the one under investigation. The question of his actual competency or incompetency must then be determined by direct and cross-examination, and the jury are the sole judges of the weight and value of such evidence. In the case, People vs. Rice (159 N.Y., 400), the Court of Appeals lays down the rule that if a man be in reality an expert upon any given subject belonging to the domain of medicine, his opinion may be received by the court though he has no license to practise; but the court further asserts that such testimony should be received with great caution, and only after the trial court has become fully satisfied that the witness is fully competent to speak upon the particular subject under investigation; where, therefore, the witness is not *prima facie* competent, because not licensed to practise medicine, it is incumbent upon the party calling him to prove him to be an expert before his testimony can be taken.

We are next led to enquire of what expert testimony consists; what the legitimate sphere of the expert is. The elementary writers upon the subject of evidence all attempt to define expert testimony, but the most lucid explanation—not definition—I have discovered, is that of Judge Werner, of the Court of Appeals, in the case of Daugherty vs. Milliken, 163 N. Y., 527. He says:

There are two classes of cases in which expert testimony is admissible, those where the conclusions to be drawn by the jury depend upon the existence of facts which are not common knowledge, and which are peculiarly within the knowledge of men whose experience and study enable them to speak with authority upon the subject. In such cases the facts are to be established by expert testimony; but the conclusions to be drawn therefrom are for the jury; this situation may be plainly illustrated in case of death from arsenical poisoning; it is the province of the analytical chemist to determine through the instrumentality of chemical analysis the presence of arsenic in sufficient quantities to produce death; that fact being established it becomes the province of the jury to determine the circumstances under which the poison was administered; of which the famous Benham case is a good illustration. In the other class are those cases where the conclusions to be drawn as well as the knowledge of the facts themselves, depend upon professional or scientific knowledge not within the range of ordinary training or intelligence. In such cases not only the facts but the conclusions to which they lead, are .to be testified to by the expert. This class may be illustrated by the frequent cases to recover for personal injury where the medical expert is called not only to state the nature of the injury, the facts regarding the same, but also to give his opinion as to its results and permanency.

But it is the same here as elsewhere, much more easy to enunciate a general rule than to apply it to individual cases; the combinations of facts and circumstances presented to the legal practitioner are as varied as the hues of the kaleidoscope. Courts accordingly find it difficult to make application of the principle stated, though apparently so plain and well defined. We, therefore, find our judges at times disagreeing as vigorously as medical experts ever do. In the case of Strohm vs. The N. Y. C. & H. R. R. R. Co., the Court of Appeals reversed the judgment of the trial court which had been affirmed by the General Term, because an expert had been permitted, under objection, to testify that "a patient sustaining such injuries, and presenting such premonitory signs, *may* develop traumatic insanity, or meningitis, or progressive dementia, or epilepsy with its results."

Judge Rapallo, in his opinion says:

Future consequences, which are reasonably to be expected to follow an injury, may be given in evidence, for the purpose of enhancing the damages to be awarded; but to entitle such apprehended consequences to be considered by the jury they must be such as, in the ordinary course of nature, are reasonably certain to ensue; consequences which are merely speculative, contingent or merely possible are not to be considered in ascertaining the damages.

The significant circumstance with regard to this decision is that Chief Judge Ruger and Judge Danforth dissented from the prevailing opinion, each holding that evidence by experts of the probable and even possible consequences of the injury, are admissible for the consideration of the jury.

In the later case of Griswold vs. The N.Y.C. & H.R.R.R. Co., Judge Finch, in his opinion, recognises the wisdom of the earlier cases which preclude the giving of evidence of future consequences which are contingent, speculative, and merely possible, but says that such rule in no manner conflicts with the principle allowing the evidence of physicians as to the plaintiff's present condition, its cause, and probable permanency. He further says:

There is an obvious difference between an opinion as to the permanency or a disease or injury already existing capable of being examined and studied, and one as to the merely possible outbreak of a new disease or suffering having their cause in the original injury; in the former case, that disease or injury and its symptoms are present and existing; their indications are more or less plain and obvious; and from their severity or slightness a recovery may be reasonably expected or the contrary; while an opinion that some new and different complication will arise, is merely a double speculation, one that it may possibly occur, and the other that if it does occur, it will be the product of the original injury instead of some new and perhaps unknown cause.

The difficulties the courts have to contend with in determining what is competent expert testimony may be best illustrated by referring to a few prominent decisions:

A physician who witnessed a homicide and who has described the entire transaction, may be permitted to testify whether the defendant's appearance and manner at the time indicated insanity. (People vs. Krist, 168 N.Y., 19.)

A physician who has made an autopsy on the body of the deceased may testify on the trial of an indictment of murder, that the injury on the head which caused the death, could not have been produced by a single blow; the matter being one of medical science and skill, involving technical knowledge of anatomy and therefore properly the subject of expert testimony. (People vs. Schmidt, 168 N.Y., 568.)

A physician who examined a woman for the purpose of testifying at the trial of an action brought by her to recover for damages for injuries sustained by her, may state what occurred in the examination, the manner of application of the tests, and the woman's acts and exclamations so far as they do not embrace statements made by her to him as her physician. (Zingrebe vs. Union Ry. Co., 56 Appt. Div., 555.)

An expert may make illustrations on a blackboard for the purpose of explaining his testimony and making it more intelligible. (McKay vs. Lasher, 121 N.Y., 477.)

An expert can not read in connection with his testimony from his own published books upon the subject. (Mix vs. Staples, 44 St. Rep., 399.)

Medical works cannot be read in evidence nor can medical experts testify to statements which such works contain. (Matter of Mason, 60 Hun, 46.)

It might be well in this connection to note, that the only use that can be legitimately made of medical works upon the examination of an expert is upon his cross-examination; he may then be asked if the author of such and such a work is an authority upon the subject of which he treats; the cross-examining counsel may then read to the witness a statement from such work and ask the witness if he concurs in such statement, the purpose being to show that the opinion expressed by the witness is at variance with that of the author.

Again, it is sometimes difficult to establish the line of demarcation, between expert and non-expert testimony. This situation was well illustrated by the case of Manke vs. People, 78 N.Y., 611. In that case one Adolph had been killed by a gunshot wound; pieces of paper were found near the scene of the homicide bearing certain marks; an expert was called to testify whether they were powder marks and whether the condition of the paper was such that, in his opinion, it was wadding fired from the gun. The trial court excluded the evidence and its conclusion in this particular was sustained by the General Term and the Court of Appeals. Justice Tallcott, presiding judge of the General Term, in his opinion said that the case was very close to the border line but, in his judgment, it was beyond the province of experts and within the province of the jury.

Now, we can easily understand how there may be very pronounced differences between the opinions of experts, without finding it necessary to impugn the integrity of either. Lawyers of extensive practice can easily comprehend this situation. Lawyers differ in their opinions regarding a novel legal proposition as radically as medical experts ever do. The lawyer who takes a case, and becomes interested in its prosecution or defence, looks upon all the facts connected with the same in the light most favorable to his contention; he examines the law with the hope—the belief—that his views are correct; he searches for authorities sustaining them; he reads every case bearing upon the subject with the view of detecting something to justify his conclusion; he often distorts the fair meaning of decisions in his zeal to make them conform to his ideas. The only impartial student of the law is one who examines his authorities in a judicial capacity, having no interest in the result, except to do what is absolutely right.

Under our present system of expert testimony the witness at once becomes interested in the result of the litigation; an attorney having an action involving the determination of some intricate expert proposition, sets out to find his expert; he realises in the first place that such expert must be a professional man of good standing and reputation whose opinion would be likely to carry weight and conviction; having selected his witness he states to him the facts in the case from his viewpoint, placing his own construction upon them; he tells the doctor that he desires to establish a certain proposition, at the same time vigorously asserting his belief in the same; he tells him that if he agrees with him and can establish such position by authorities he desires to use him as a witness, and that he will properly compensate him. The very moment the doctor consents to such arrangement he becomes as much interested in the final result of the controversy as the lawyer himself; he begins thinking along the lines indicated; if he has doubts he reasons them away; he consults his authorities culling out the portions sustaining his contention and discarding those which do not; he becomes imbued with the belief, perhaps unconsciously entertained, that he has a direct personal interest in the litigation.

The criticism upon all this is not so much upon the doctor as upon the system; just as long as our present system prevails so long shall we encounter the conditions to which I have referred; as long as parties or their attorneys are permitted to select their own experts, schooling them in advance along the lines desired, so long will we find this radical conflict in the opinions of the experts on opposite sides of a case and upon propositions where it would seem to the ordinary observer that doctors should agree.

The question naturally arises, What is the remedy? How should our present system of practice be modified so as to eliminate these difficulties? This is, of course, the all important consideration, but I must confess that the solution of this problem is not free from difficulty. Several methods have been proposed by lawyers who have given this matter careful consideration and yet no plan has been suggested which meets the requirements. The system prevailing in this country and in England has often been compared with that in vogue in France and Germany, where the expert is designated by the court and so far as his functions in a particular case are concerned he becomes a part of the judicial system. The comparisons made between these two systems are usually in favor of the French and German methods, and yet they are not free from fault; they complicate judicial procedure, impose upon courts the extra responsibility of selecting the expert, and subject the court to the criticism of having made a selection favorable to the successful party.

It has also been proposed that a state commission of experts should be created, a member of which should attend and testify at the trial of cases involving expert questions. A little consideration, however, will show how intensely impracticable is such a scheme; expert testimony covers the range of every known science and every skilled occupation; it would be utterly impossible to establish a commission, no matter how large, competent to deal with all the subjects which claim the attention of the expert. As a result, a separate commission would be required for almost every field of human investigation and the expense of maintaining the same would be beyond computation.

A condition should be created by public sentiment if possible or by actual legislation if necessary, making the existence of the professional expert witness an impossibility. I do not wish to be understood as casting any reflection upon any physician because he is frequently called as an expert witness, for I regard such a situation complimentary to him. By the term "professional expert witness," I mean the one who makes his professional opinion an article of merchandise in the legal market ready to be sold to the highest bidder. This class of witnesses is by no means a myth; the country is filled with them; they are usually ingenious, crafty and unscrupulous; having sold an opinion, they become solicitous to have such opinion prevail, whether right or wrong, in order to enhance the market value of their opinion when needed again; the innocence of a defendant is of no consequence where the opinion of the professional is assailed; to gratify his professional pride and ambition he is willing to consign even the innocent to prison or the electric chair.

I was on one occasion connected with the contest of the probate of a will, where it was claimed that the signature to the will was a forgery; a professional expert on handwriting was consulted who offered to demonstrate the forgery before the jury, making his fee of $500 contingent upon the result; the contestant, declining such offer, was met upon the trial of the case, with the evidence of this same witness with a detailed explanation amounting to almost a mathematical certainty that the signature to such will was genuine. I would as soon rely upon the opinion of Judas Iscariot as to the efficacy of the vicarious atonement, as upon the opinion of such a witness. The achievements of this class of experts are not only repugnant to one's sense of humanity and justice, but are also largely responsible for the adverse criticisms passed upon your profession. It is your duty, as representatives of one of the most magnificent of all the learned professions, to zealously guard the honor and maintain the dignity of such profession; you can best accomplish this result by an extermination of the class of professional vipers to which I have referred.

Then, again, there should be a radical modification of our present method of using hypothetical questions in the examination and cross-examination of witnesses; the practice which has developed and so extensively prevails along this line has become simply farcical, where not absolutely dangerous. The proposition is axiomatic that a hypothesis founded on fallacy can never evolve the truth; if the facts in a case were all conceded then a hypothesis based upon such admitted facts might throw some light upon the controversy; but when the facts are controverted, as they usually are in litigations, this practice of examination by hypothesis is confusing, misleading and by no means conducive to honest and equitable results. The purpose of the examiner in framing his hypothetical question is two-fold; first, to so state it as to insure a favorable answer; and, second, to so disguise the assumed facts that the jury may consider the answer alone. Under our present system of evidence the attorney calls his expert, reads to him a complicated hypothetical question covering the facts as such attorney claims them to be, and asks for the witness's opinion. The opposing counsel calls his expert, frames his hypothetical question in accordance with his views of the facts and asks the opinion of the witnesses. The argument is then made by each counsel to the jury that the opinion of the opposing expert is worthless because based upon facts not established by the evidence; consequently the jury either ignores the entire proceeding or, what is more likely, derives impressions therefrom radically and fatally wrong.

The vice of the hypothetical question as used in our present practice is that such question is usually predicated upon isolated or disconnected facts or statements; it is a matter of common observation that the rationality or criminality of any particular act or statement is not determined so much from such act or statement independently considered, as from what immediately preceded or followed it; no reliable estimate of one's mental condition can be predicated upon isolated and independent acts; it is the entire line of conduct, each act being examined with reference to what preceded and that which followed it, which indicates whether the man is controlled by the dictates of sense and reason, or by the illusions of a disordered mind or imagination.

In the Matter of Jones, it was shown that the testator was accustomed to speak of his estate with great exaggeration, saying that he had more property than any one knew of, sufficient to make all his relatives rich; he asked the witness Brown to give him his daughter, saying that he wanted her for his wife; that he would make her a necklace of twenty dollar gold pieces that would go around her neck and reach to the ground, one that she could jump through. The evidence showed that he was of a licentious nature, vulgar and indecent in conversation, passionately fond of narrating his amorous exploits, speaking of his liaisons publicly and boastingly, and the court, in deciding the case, said that the inference was fairly justified by the evidence that the testator was either a notorious romancer or a veritable Don Juan.

In the Matter of Vedder, it was shown that the testatrix had a firm belief in witches and witchcraft; that she entertained various hallucinations such as that she had seen and conversed with Jesus; that if live coals and a red garter were put under a churn, the butter would come; that it was impossible to keep her horses in good condition because the witches rode them at night.

In the case of VanGuysling vs. VanKuren, it appeared that the testatrix believed she was tormented by witches and spooks which kept her awake at night; she became violently enraged when told there were no witches and spooks; she imagined she saw things which did not exist, and described visions she had while sleeping; she used her fingers in place of knife and fork, and her eyes were frequently wild and glaring. In either of these cases, a pronounced opinion of mental incompetency from honest experts would have been elicited, in answer to a hypothetical question based solely upon these particular acts or lines of conduct, and yet in each case the will was admitted to probate and the testamentary capacity of the testator established because such acts and statements, when viewed in the light of the general line of conduct of the testator, were seen to be of but little importance.

In a will contest recently tried in Cattaraugus County it appeared that the will offered for probate was executed on the 21st day of May, 1901; on the day following its execution the testatrix inquired of a neighbor how much arsenic would be required to take one's life; she had frequently expressed a determination to commit suicide; on the 29th of May, she was found hanging by the neck from the rafter of her barn, having committed suicide. A physician of that county, who had been well acquainted with the testatrix for many years, and who had attended her and had had business transactions with her, pronounced her entirely sane at the time of the execution of the will. I quote from the cross-examination of the same witness:

Q. Do you regard suicide as evidence of insanity?

A. I do.

Q. In your opinion was this woman insane at the time she committed suicide?

A. I think she was.

Q. Do you regard a settled determination and design to commit suicide as evidence of insanity?

A. I do.

Q. Now, assuming that this woman committed suicide by hanging on the 29th of May; that on the 22d of the same month she inquired as to the amount of arsenic required to take one's life; that she had often expressed a determination to take her own life, what in your opinion was her mental condition on the 21st, the day of the execution of the will.

A. If I were to base my answer upon the facts stated in the hypothetical question, I should say she was insane, but I am unable to reconcile my opinion as an expert based upon such a hypothesis, with my opinion based upon personal observation of this woman for many years before her death and even after the date of the will.

These illustrations are entirely sufficient to establish the force of the criticism I have made upon evidence of this character. It is true that the total abolition of hypothetical questions would effect a very radical change in our present system, because it would eliminate all medical expert testimony except where the witness testified from personal observation and examination. The witness having examined the patient and detailed the results of such examination and the facts thereby disclosed, might then express his professional opinion regarding the same; such an opinion would be valuable because based upon actual facts coming under the personal observation of the witness, and not upon some vague and confusing hypothesis. In most cases no hardship could result from the application of such a rule, because under existing laws the opposing party has the right of personal examination of the injured party by his own expert, thereby qualifying him to speak from actual observation.

Again, the compensation of experts should be in some manner regulated and limited; every expert when called upon to testify as such should receive fair and reasonable compensation for his time and services; such compensation should be as much as he would ordinarily receive for the same amount of time and service in the usual performance of the duties of his profession. Compensation to that extent is just and honest; but every dollar paid an expert in excess of such a sum for the purpose of stimulating his zeal or biasing his judgment is decidedly unfair and dishonest. An established rate of compensation, would relieve your profession from much embarrassment arising from the necessity of making a contract as to your fees each time you are called as an expert, or from calling for the protection of the court when some attorney attempts to obtain expert testimony from you for ordinary witness fees.

The conclusion reached from this line of thought is, that the defects existing in our present system of expert testimony are more the fault of the system than of the doctor. As a rule, the doctor is honest and conscientious; give him an opportunity of expressing what he may know unfettered by technicalities and we need have no fears of medical expert testimony. History records the fact that after the death of Esculapius, he was placed by the Greeks among their demigods and the Romans constructed a temple upon the island of the Tiber to perpetuate the fame of the "Father of Medicine." From that day to this mankind has honored and respected this profession. We trust the doctor in our most trying ordeals; when the messenger of death is hovering near and we can almost hear the dread summons to "join the innumerable caravan," our faith, our hopes are all centered in the skill, intelligence and ability of the doctor; we certainly can rely upon his integrity where our welfare as litigants is involved.

